# Leveraging Artificial Intelligence (AI) Based Algorithm for Accurate Estrogen Receptor (ER) and Progesterone Receptor (PR) Analysis in Breast Cancer Diagnostics: Potential to be a Crucial Aid in Routine Workflow

**DOI:** 10.12688/f1000research.175934.2

**Published:** 2026-08-03

**Authors:** Kanthilatha Pai, Brij Mohan Kumar Singh, Chethana Babu Udupa, Madhavi Pai, Vani Verma, Sumit Jha, Kiran Aatre, Purnendu Mishra, Vishwapriya Mahadev Godkhindi, Swati Sharma, Gursevak Singh, Shubham Mathur, Akash Modi, Rajiv Kumar

**Affiliations:** 1 Dept of Pathology, Kasturba Medical College, Manipal Academy of Higher Education, Manipal, Karnataka, 576104, India; 2 Applied Materials India, Bangalore, Karnataka, 560066, India; 3 Mangalore Institute of Oncology, Mangaluru, Karnataka, 576104, India

**Keywords:** breast cancer, biomarker expression, Estrogen receptor, Progesterone receptor, algorithm, manual

## Abstract

**Background:**

Scoring of estrogen receptor (ER) and progesterone receptor (PR) expression in breast cancer is critical for identifying patients who would benefit with hormonal therapy. Since manual scoring of immunohistochemistry (IHC) is influenced by pathologist experience, fatigue, inter-observer variability, and subjectivity, artificial intelligence (AI)–based algorithms, trained on large datasets can aid to improve diagnostic accuracy.

**Methodology:**

This study evaluated an AI-based algorithm for ER and PR IHC scoring in 297 ER and 293 PR cases of invasive breast carcinoma and compared the scores with that of pathologists (two senior and two junior) A pre-trained automated algorithm (Mimansa) identified region of interest and provided the scoreswhich was compared with the consensus score of pathologists-ground truth(GT).Concordance was evaluated using Cohen’s kappa and F1 score.

**Results:**

For ER IHC, GT scores included 169 strong positive, 31 low positive, and 98 negative cases. Agreement with GT was 99% and 98% for senior pathologists, 97% for the AI algorithm, and 95% and 93% for junior pathologists. The algorithm correctly classified all strong positive cases but showed discordance in 16 low-score cases, with four false negatives and ten false positives. Notably, it identified two true positive cases missed by all pathologists.

For PR IHC, agreement rates were 98% and 97% for senior pathologists, 92% for the algorithm, and 93% and 91% for junior pathologists. The algorithm achieved perfect accuracy in strong positive cases but produced 16 false negatives and eight false positives among low-score cases. Cohen’s kappa values were 0.91 (ER) and 0.84 (PR).

**Conclusion:**

The AI algorithm demonstrated high concordance with expert consensus, performing comparably to senior pathologists and outperforming junior pathologists in several metrics. It shows promise as a supportive second-reader tool, particularly in low-positive cases where diagnostic errors may significantly impact patient management.

## Introduction

The presence and degree of Estrogen receptor (ER) and Progesterone receptor (PR) expression hold significant importance in both prognostic evaluation and selection of the most suitable treatment strategy in patients with breast cancer. ^1^ Studies highlight that positive receptor status often correlates with better outcomes and responsiveness to hormone therapy, improving survival rates. ^2^

Interpreting ER and PR expression in breast cancer poses challenges owing to its subjective nature, which is influenced by factors such as pathologist experience, training, and workload fatigue. ^3– 5^ Recent advancements suggest that Artificial Intelligence (AI) can enhance the accuracy and reproducibility of these assessments. ^5, 6^ Studies show AI’s potential in standardizing receptor quantification and improving diagnostic precision. ^7^

While there have been several studies reporting on the development of individual AI models for the assessment of Her2, ER/PR in breast cancer, which have demonstrated good accuracy and reliability, their adoption has been limited because of several factors such as cost, lack of validation, and integration into routine pathology workflows. ^8, 9^

In this study, we conducted a comparative analysis of scores between the algorithm, junior, and senior pathologists in assessing the scores of ER and PR expressions in breast carcinoma to validate the performance of our in-house built algorithm (Mimansa) and its potential for incorporation into the workflow to improve diagnostic accuracy.

## Materials and methods

We conducted a comprehensive search of our pathology database to retrieve 300 surgical pathology cases to include all cases of invasive breast carcinoma of all types diagnosed from March 2020–March 2021, and study period started from June 2021 after obtaining Institutional ethics committee approval. The study incorporated a mix of slides from trucut biopsy and resection specimens to validate the algorithm across different sample types.

In our study, we obtained hematoxylin and eosin (H&E)-stained slides and immunohistochemically (IHC) stained slides for ER and PR. We excluded slides of poor quality, those with bubbles, and those that faded. Ultimately, we included 297 ER IHC slides and 293 PR IHC slides, along with their corresponding H&E slides, to ensure correlation and validation of the algorithm’s performance.

### Estrogen receptor (ER) and Progesterone receptor (PR) immunohistochemistry

ER expression was assessed using formalin-fixed paraffin embedded (FFPE) tissue sections (ischemic time < 1 h and fixation time between 3 and 36 h) by immunohistochemistry (IHC) using an anti-ER antibody (clone EP1 mouse monoclonal antibody; Dako) and anti-PR (Clone PgR636 mouse monoclonal antibody; Dako), and staining was performed using an automated IHC stainer (Ventana Benchmark XT, Ventana Medical Systems Inc., Tucson, AZ, USA). ER and PR expression was scored using the Allred scoring system as shown in Table 1.

**Table 1.** Allred score for ER and PR expression.

Percentage score		Intensity score	
Score	Percentage of stained cells	Score	Intensity of staining
0	No cells are ER/PR Positive	0	Negative
1	<1% cells are ER/PR Positive	1	Mild
2	1–10% are ER/PR Positive	2	Moderate
3	11–33% are ER/PR Positive	3	Strong
4	34–66% are ER/PR Positive		
5	67–100% are ER/PR Positive		

Allred Score = Percentage score + Intensity score.

Algorithm development

*Methodology*

In this paper, we present a fully automatic multi-class tissue segmentation in-house built algorithm (Mimansa) that classifies tumors as well as other tissue regions, such as acini, ducts, and DCIS, into fine-grained segmentation maps of histopathology images. The proposed model works with multiple stains of breast IHC, including and not limited to nuclear (ER, PR) stains. The model was built using 10 million patches extracted from 513 WSI from multiple data sources and multiple scanners to account for strain and scanner illumination variations. The 513 WSI were used for model development (training, validation and testing). The 297 ER and 293 PR cases were separate data set that were used to test the developed algorithm.

For the ground truth, because complete slide annotation is very difficult and time-consuming, a selective region-wise annotation technique was utilized. The pathologists selected 2–3 mutually exclusive non-similar small regions and annotated them completely with multiple labels, including tumor, normal, stroma, acini, duct, blood vessels, and DCIS. Annotations for bad regions such as Folds, Artifacts, bubbles, and out-of-focus areas were also performed, and the patches were extracted from the annotated regions.

*Class imbalance*

In clinical samples of WSI, tumor regions are far fewer than normal tissue regions and the background. Using filtration techniques, the non-tissue area and background were ignored. Because the majority of the annotated tissue regions were either Tumor or Normal/Stroma tissues, all additional regions (blood vessels, DCIS, acini, ducts, folds, skin, and Unknowns) were classified into a section called others. A data generator pipeline was introduced to ensure that all batches had a similar class distribution of 2:1:1 (tumor: normal: others). The algorithm also accounts for pixel-level imbalances of the dataset using a set of normalized dynamic weights.

*Data augmentation and final model*

The AI pipeline employed a two-stage architecture. A U-Net-based segmentation model was used initially to identify tumour regions within whole-slide images, restricting subsequent analysis to regions of interest. Nuclei detection and classification within these tumour regions were then performed using a customized Mask R-CNN model. The algorithm was trained on manually annotated image patches generated by expert pathologists, with extensive data augmentation (rotation, translation, shearing, cropping, and colour perturbation) were used to improve robustness. Performance was evaluated at both the cell level against expert annotations and the slide level using independent datasets. The two-stage approach reduced the average processing time from approximately 30 to 12 minutes per slide while maintaining high reproducibility, with approximately 99% agreement across repeated analyses.

The images were adjusted for visual (brightness and contrast), color, and texture differences. Augmentation techniques were used to enhance the dataset.

To compensate for the manually annotated dataset, the Phase-1 model learns basic nuances such as cell structures, background, regions to ignore, different tissue types, and staining colors. However, this model cannot be scaled to different data sources, scanner variations, and similar looking tissues such as DCIS and ACINIs, which look like tumors and have smaller FOV. In Phase-2, works on increasing augmentations and predictions. This allows for a tighter thresholding, thereby increasing the accuracy of the model.

### Whole slide scanning and analysis

**Figure 1.**
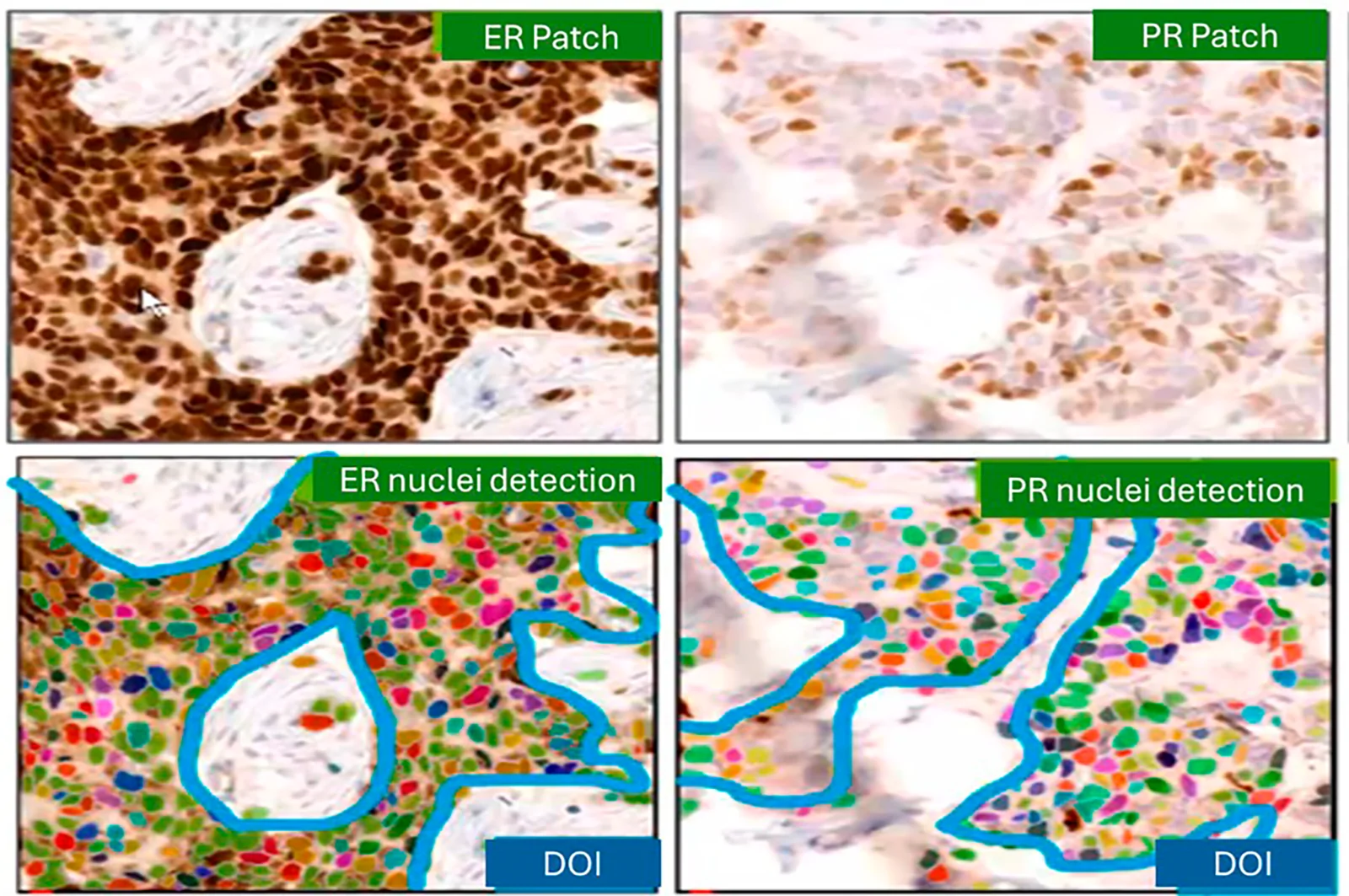
Photomicrograph shows identification of tumour regions (region of interest) and nuclei detection using multiple colours by algorithm. This image is output generated by the algorithm, which was developed by training AI tool (Mimansa) by pathologists and software engineers who are listed as authors to detect and represent hormone receptor staining patterns.

**Figure 2.**
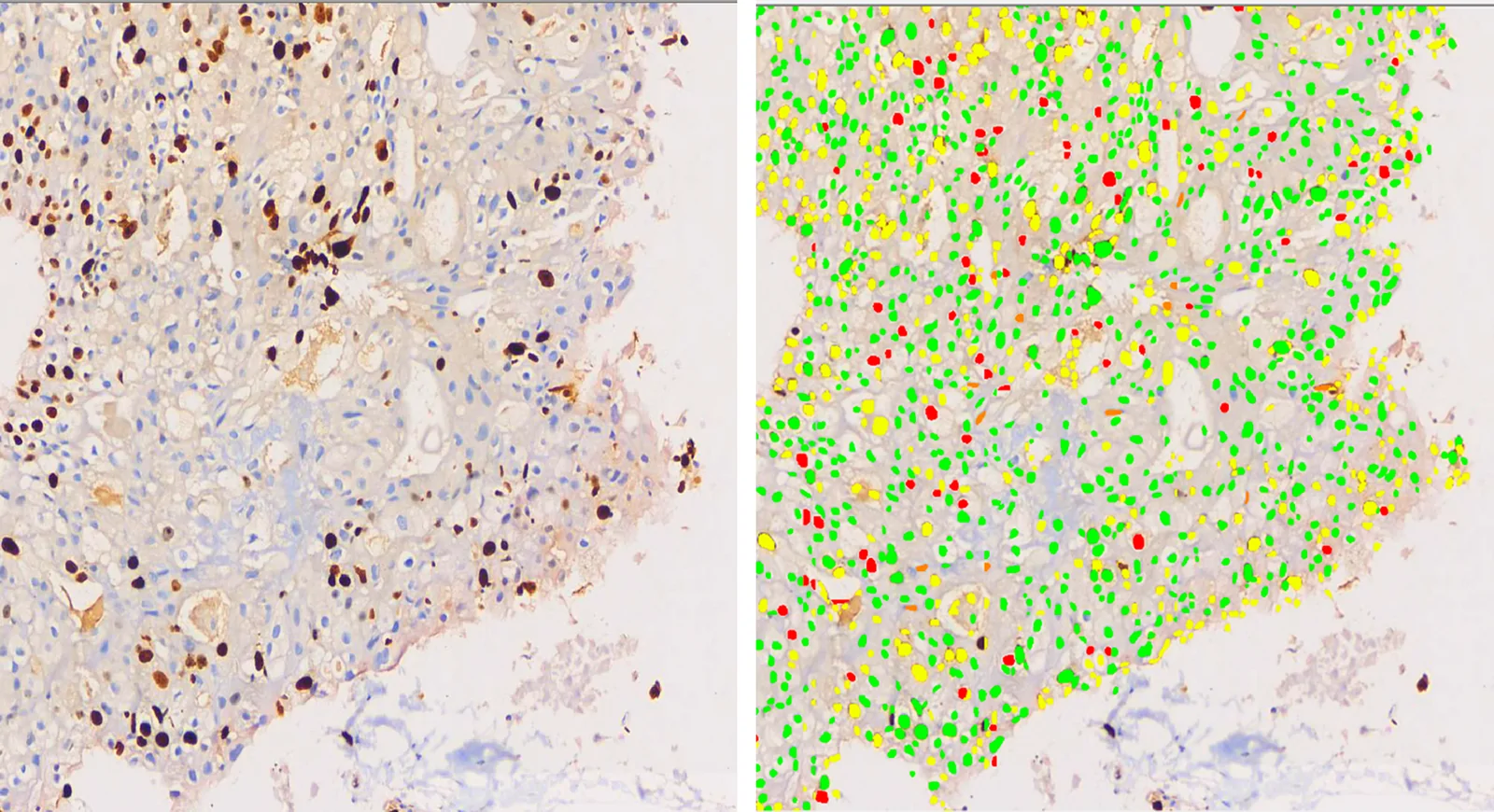
Photomicrograph shows IHC stained ER slide on left and corresponding detection and classification of nuclei as negative (green), weak positive (yellow), moderate positive (orange) and strong positive (red) by algorithm. This image is output generated by the algorithm, which was developed by training AI tool (Mimansa) by pathologists and software engineers who are listed as authors to detect and represent hormone receptor staining patterns.

The slides were scanned using Morpholens 6 T at 40X magnification and uploaded to a cloud-based platform. The in-house built-in algorithm (Mimansa) was run to score each case, providing the proportion of positively stained tumour cells for ER and PR and the intensity of positive cells to generate a total score according to the Allred scoring system ( Figures 1 and 2). Manual scoring of ER and PR IHC of the above slides was independently performed by four pathologists: two senior pathologists with over 15 years of experience in breast pathology (Sr Path 1, Sr Path 2) and two with less than three years of experience (Jr Path 1, Jr Path 2). The ER and PR scores were dichotomized, with ER/PR positivity recognized at an Allred Score cut-off of 3. Using this cut-off, scores of 0 to 2 were considered negative for ER/PR and not actionable, while scores of 3 to 8 were regarded as positive and suitable for hormonal therapy according to the American Society of Clinical Oncology/College of American Pathologists guidelines. Any discrepancy between the scores of pathologists resulting in an actionable outcome (cut-off score of 3) that would affect therapeutic decision was reviewed to obtain an initial consensus score.

The algorithm was run on the digitized slides to obtain Allred scores for ER and PR similar to that of the pathologist’s score. The algorithm score was compared with the initial (pathologist) consensus score, and any discordance was reviewed to obtain a final consensus score (ground truth). The consensus score was obtained by joint review of all discordant cases by the four pathologists using a penta-head microscope. Discrepancies were resolved through discussion to obtain a final consensus score (ground truth). No third independent pathologist was involved, as all disagreements were resolved through this consensus review.

**Figure 3.**
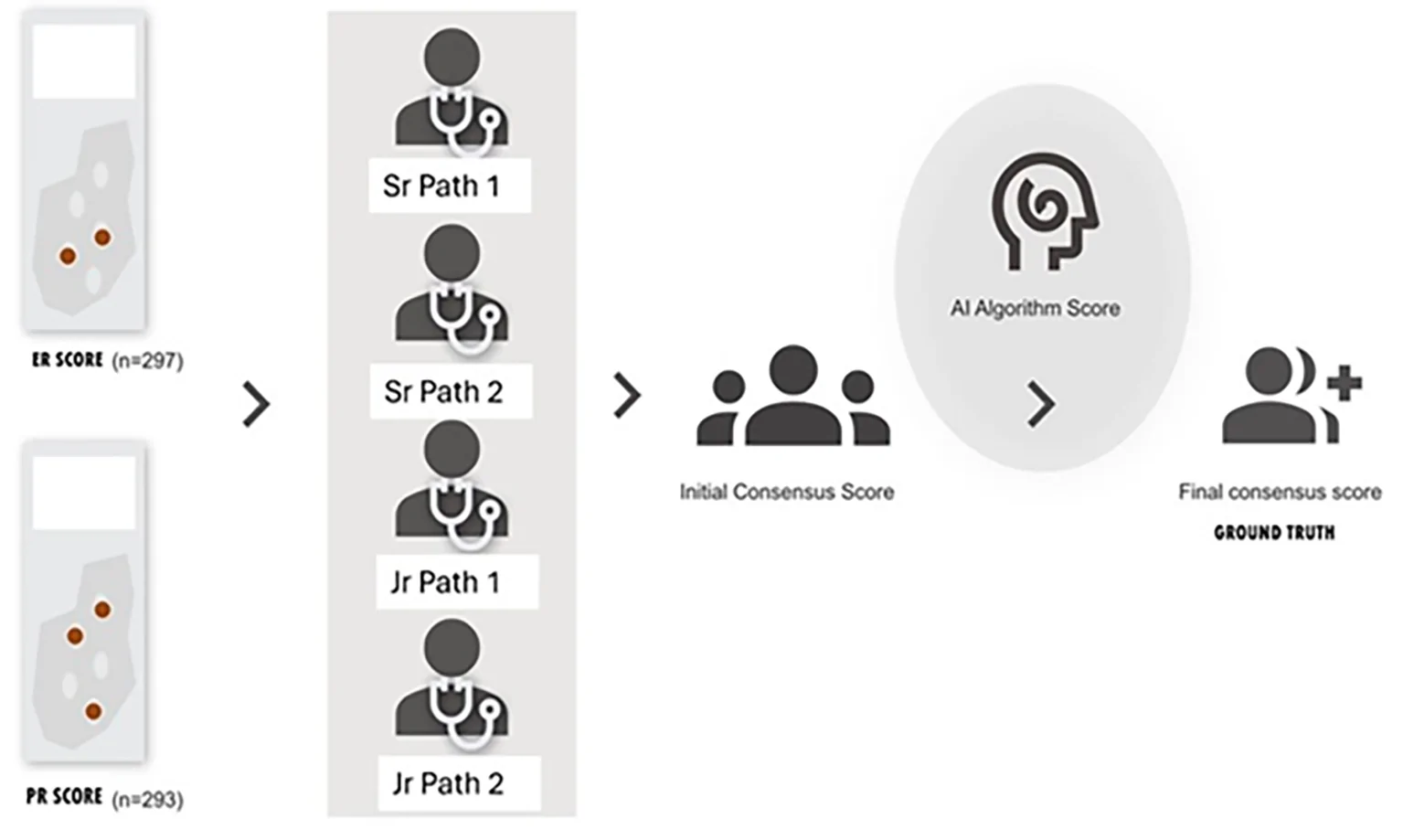
Study design to evaluate the performance of AI based algorithm scores with pathologists’ score: The algorithm score was compared with the initial (pathologist) consensus score by pathologist’s and any discordance was reviewed to obtain a final consensus score (ground truth). The Google material design icons were used for the figures.

The study design is depicted in Figure 3. ER and PR scores were further classified into three groups: 0–2 as negative, 3–5 as low positive and 6–8 as strong positive. The agreement and reliability between the raters and the AI-based algorithm were statistically analyzed.

### Statistical analysis

Data were analyzed using IBM-SPSS Statistics for Windows version 23.0 (Armonk, NY, IBM Corp). Categorical data were expressed in terms of proportions and percentages. Inter-rater reliability analysis was performed using Cohen’s Kappa with significance testing and confidence intervals to see the agreement of the algorithm with ground truth for ER/PR scores across different score ranges as well as three groups: negative, low positive, and strong positive scores. Pearson’s correlation coefficient was used to evaluate the agreement of scores generated by the algorithm and other raters, which included senior pathologists and junior pathologists with the ground truth (final consensus scores).

## Results

The age of the patients ranged from 18 to 79 years. Trucut biopsies formed majority of cases (60.60%).

The most common histopathological diagnosis was Invasive ductal carcinoma of no special type. Luminal type showing either ER and/or PR positivity accounted tor 82.15% of the cases. Table 2 shows the pathological features of the 297 cases of breast carcinoma analysed in this study.

**Table 2.** Shows the demographic features of the cases included in the study (N = 297).

		Number	Range/percentage
Age			18–79 years
Specimen type	Trucut biopsy	180	60.60%
	Resection	117	39.39%
Histological type	Invasive ductal carcinoma, NST	218	73.40%
	Invasive lobular carcinoma	21	7.07%
	Others	58	19.53%
Molecular subtype	Luminal type (ER and or PR positive)	244	82.15%
	**Her 2 status**		
	Negative (0,1+)	237	79.80%
	Equivocal (2+)	25	8.42%
	Positive (3+)	35	11.78%
	Triple negative	53	17.85%

**Figure 4.**
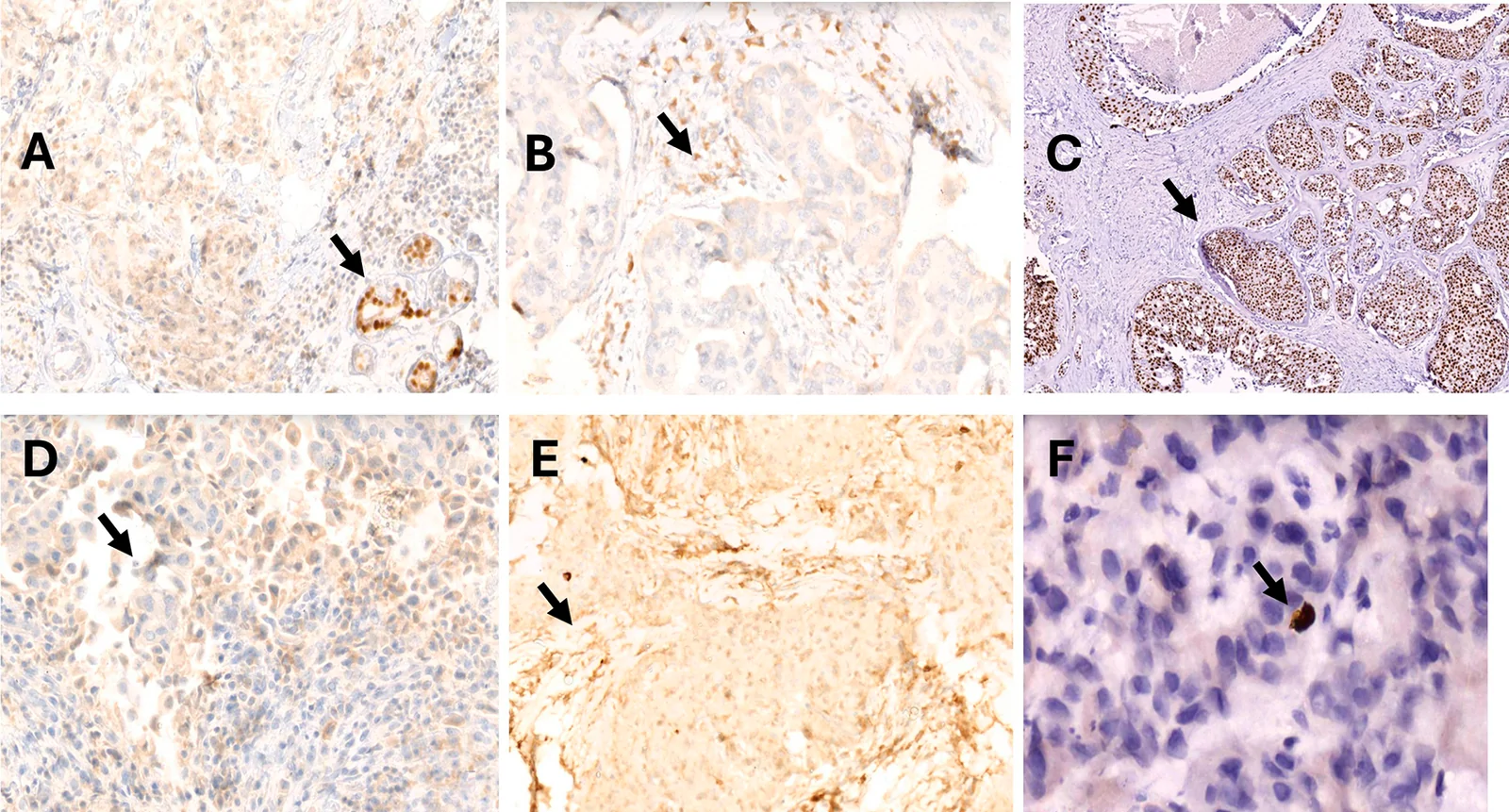
Photomicrograph shows reasons for misinterpretation of ER/PR expression by algorithm giving false positive scores (marked with arrow) A) Positive staining in normal interspersed breast acini, while tumor cells are negative B) positive staining in stromal cells while tumor cells are negative C) Ductal in situ component positive while invasive tumor negative D) non- specific cytoplasmic staining while nuclei negative E) non-specific background staining F) brown stain particles while tumour cells negative.

Table 3 reveals the performance of algorithm for ER and PR expression.and the reasons for mis-interpretation. There was concordance of 93.93% with ER expression, while slightly lower at 87.37% for PR expression scoring by algorithm when compared with ground truth. The reasons for misinterpretation are listed in Table 3 and in Figure 4.

**Table 3.** Shows the agreement of ER and PR scores between algorithm and ground truth and the reason for mis-interpretation.

Comparison of algorithm scores with ground truth	ER scores		PR scores		Reason for mis-Interpretation
	Total No (297)	Percentage	Total No (293)	Percentage	
Concordant	279	93.93%	256	87.37%	
Discordant (False positive)	09	3.03%	19	6.48%	➢ Positive staining of normal breast acini ➢ Ductal carcinoma in situ positive, while invasive tumour negative ➢ Tissue folds ➢ Stain particles ➢ Non- specific staining of stromal cells ➢ Non- specific staining of cytoplasm of tumour cells
Discordant (False negative)	07	2.35%	18	6.14%	< 10% cells missed by algorithm
Discordant (True positive)	02	0.67%	---	----	Review of 2 cases of ground truth score of 0 and positive by Algorithm was confirmed to be low positive (Score 3–4). All the pathologists had missed few positive (<10%) cells in their interpretation which was detected by algorithm

There were no statistically significant differences in ER and PR scores between the trucut biopsy and resection specimens.

Tables 4– 6 reveal Algorithm performance for different scores, low positive and strong positive scores as well as comparison with senior and junior pathologists for ER expression.

**Table 4.** Algorithm agreement with ground truth for Allred ER scores (0–8) N = 297.

ER	Conditional	Kappa	Asymptotic			Asymptotic 95% Confidence interval	
Scores	Probability		Standard error	Z	Sigma	Lower bound	Upper bound
0	.879	.821	<015	54.803	<001	.792	.850
2	.107	.86	<015	5.76	<001	.57	.116
3	.355	.325	<015	21.671	<001	.295	.354
4	.275	.246	<015	16.439	<001	.217	.276
5	.302	.275	<015	18.362	<001	.246	.304
6	.251	.213	<015	14.23	<001	.184	.243
7	.277	.181	<015	12.106	<001	.152	.211
8	.780	.683	<015	43.578	<001	.623	.623

**Table 5.** Algorithm performance in negative, low positive (3-5) and positive (5-8) groups with ground truth for ER expression, N = 297.

ER	Conditional	Kappa	Asymptotic			Asymptotic 95% Confidence interval	
Scores	Probability		Standard error	Z	Sigma	Lower bound	Upper bound
Negative (0–2)	.929	.891	<015	59.454	<001	.861	.920
Low positive (3-5)	.636	.587	<015	39.158	<001	.557	.616
Strong positive (6-8)	.963	.921	<015	61.48	<001	.892	.950

**Table 6.** Shows the inter-item correlation matrix for ER scores, comparing the algorithm (AI) and various pathologists against the ground truth.

	Ground truth	AI (algorithm)	Sr Path1	Sr Path 2	Jr Path 1	Jr Path 2
Ground truth	1.000	.937	.992	.976	.966	.958
AI (algorithm)	.937	1.000	.933	.921	.918	.912
Sr Path 1	.992	.933	1.000	.974	.966	.957
Sr Path 2	.976	.921	.974	1.000	.963	.952
Jr Path 1	.966	.918	.966	.963	1.000	.972
Jr Path 2	.958	.912	.957	.952	.972	1.000

For ER expression, the ground truth (GT) revealed 169 strong positive, 31 low positive, and 98 negative cases. Concordance with GT was highest among senior pathologists (99% and 98%), followed by the AI algorithm (97%), and slightly lower for junior pathologists (95% and 93%). The algorithm was particularly accurate in correctly classifying all strong positive cases; however, discordance was observed in 16 low-score cases, including four false negatives and ten false positives. Importantly, the algorithm correctly identified two true positive cases that were missed by all pathologists.

Tables 7– 9 reveal Algorithm performance for different scores, low positive and strong positive scores as well as comparison with senior and junior pathologists for PR expression.

**Table 7.** Algorithm agreement with ground truth for All red PR scores (0–8) N = 293.

PR	Conditional	Kappa	Asymptotic			Asymptotic 95% Confidence interval	
Scores	Probability		Standard error	Z	Sigma	Lower bound	Upper bound
0	.875	.776	.015	51.474	<001	.747	.806
2	.131	.104	.015	6.913	<001	.075	.029
3	.274	.237	.015	15.718	<001	.208	.134
4	.362	.313	.015	20.726	<001	.283	.267
5	.319	.276	.015	18.326	<001	.247	.342
6	.347	.302	.015	20.024	<001	.272	.306
7	.297	.240	.015	15.904	<001	.210	.269
8	.768	.703	.015	46.584	<001	.673	.732

**Table 8.** Algorithm performance in negative, low positive (3-5) and positive (5-8) groups with ground truth for PR expression N = 293.

ER	Conditional	Kappa	Asymptotic			Asymptotic 95% Confidence interval	
Scores	Probability		Standard error	Z	Sigma	Lower bound	Upper bound
Negative (0–2)	.910	.829	.015	54.648	<001	.799	.858
Low positive (3-5)	.618	.534	.015	35.199	<001	.504	.563
Strong positive (6-8)	.906	.856	.015	56.477	<001	.827	.886

**Table 9.** Shows inter-item correlation matrix for PR scores, comparing the algorithm (AI) and various pathologists against the ground truth.

	Ground truth	AI (algorithm)	Sr Path 1	Sr Path 2	Jr Path 1	Jr Path 2
Ground truth	1.000	.887	.984	.980	.932	.937
AI (algorithm)	.887	1.000	.875	.891	.889	.879
Sr Path 1	.984	.875	1.000	.964	.921	.931
Sr Path 2	.980	.891	.964	1.000	.931	.932
Jr Path 1	.932	.889	.921	.931	1.000	.937
Jr Path 2	.937	.879	.931	.932	.937	1.000

The above results for PR immunohistochemistry reveals 98% and 97% agreement for senior pathologists, 92% for the algorithm, and 93% and 91% for junior pathologists with ground truth. The algorithm for PR expression also achieved perfect concordance in strong positive cases but showed reduced performance in low-score cases, resulting in 16 false negatives and eight false positives. Cohen’s kappa coefficients demonstrated excellent agreement for ER (κ = 0.91) and strong agreement for PR (κ = 0.84).

## Discussion

Artificial intelligence (AI) has increasingly been applied across various domains of medical imaging, including image reconstruction, processing, analysis, and predictive modeling. ^10^

In breast cancer, AI technologies have been widely adopted for early detection through AI-assisted radiology and for diagnostic assessment and biomarker evaluation in digital pathology. ^11^ Appropriate image preprocessing and augmentation strategies have been shown to improve feature extraction and enhance the performance of deep learning models in medical image analysis. ^12^

Assessing estrogen receptor (ER) and progesterone receptor (PR) status by immunohistochemistry in breast cancer is essential, as it guides treatment decisions and predicts therapeutic responses. ^13– 15^ However, the reliability of assay results depends on both the consistency of assay performance and the accuracy of its interpretation. ^16^ Automated immune-stainers with high reproducibility, combined with whole slide digitalization and dedicated software helps in objective image quantification through color and intensity segmentation, allowing for unbiased scoring. ^17^

Several studies have validated the performance of automated scoring methods against manual assessment of estrogen receptor (ER) and progesterone receptor (PR) expression in breast cancer and have shown a strong correlation between automated image analysis and manual scoring techniques, suggesting that automated methods can serve as reliable alternatives. ^17– 19^ Some models were based on tissue microarrays, while others utilized whole slide images for analysis. ^17, 20, 21^ Some of the algorithms used required training and inputs from pathologists, while others have used algorithms that do not require supervision. ^22, 23^ Automated or digital image analysis has several advantages, such as reducing the bias in sampling, reducing inter- and intra-reader variability, providing more consistent reporting, and significantly reducing pathologists’ workload. ^24^

In our study, we demonstrated that the algorithm developed (Mimansa) was able to detect the relevant tumor regions from WSI, quantify immunohistochemical expression, generate ER and PR scores, and effectively replicate the ER and PR scores produced by pathologist visual scoring. Our study showed good agreement of the AI algorithm in assessing ER and PR scores with the ground truth, which was slightly lower than that of pathologists. There was no statistically significant difference between the scores of senior and junior pathologists as well as the algorithm. The overall agreement between the ER score and ground truth was 93.93%, and the PR score was 87.37%. Similar results were noted in a study by Jung et al., which showed 93% concordance for ER expression (197 cases) and 89.4% (199 cases) for PR expression by the algorithm. ^25^ Shafi et al. observed 93.85 concordance for ER expression in their study on 97 cases ^26^ and the Pearson correlation coefficient (PCC) score in our study was 0.937 and 0.887 for ER and PR, respectively, which was similar to the study by Bankhead et al., which showed a PCC of 0.908 and 0.862 for ER and PR, respectively. ^27^ Sharangpani et al. found an agreement of 85% and 81% between the automatic determination of positivity/negativity of ER and PR-stained cells with manual scoring. ^18^ Gokhale et al. reported 95% concordance between automated and manual scoring, while Mofidi et al. demonstrated a highly significant correlation (r2 = 0.844) between digital and manual ones. ^7, 17^

In our study, the algorithm demonstrated excellent agreement for negative (0–2) and strong positive (6–8) groups, with kappa values of .891 and .921 for ER expression and .829 and .856, respectively, for PR expression, and moderate agreement for the low positive (3–5 scores) group with a kappa of .587 for ER expression and .534 or PR expression, suggesting the need for refinement of the algorithm to identify low positive (3–5) scores. We identified reasons for the inaccuracies in this group with false-positive scores due to misinterpretation that occurred due to interspersed positive staining of normal breast acini, ductal carcinoma in situ component when the invasive component was negative, tissue folds with brown staining, stain artifact clumps on the tissue, non-specific staining of stromal cells, and cytoplasm of the cytoplasm of tumor cells. Shafi et al. also reported three cases of false-positive ER expression by DIA, which was mainly due to intermixed benign glands in the tumor area, ductal carcinoma in situ (DCIS) components, and tissue folding. ^24^ However, these false-positive scores in our case could be mitigated by incorporating an option in the algorithm to manually exclude areas such as tissue folds, normal breast acini, etc. before re-running the analysis. Such misclassification errors occurring from poorly stained samples or samples of bad quality can be overcome by the ability of digital image analysis to reclassify or drop individual detected objects and recalculate the software provided results. ^28^

We noticed 7 false-negative cases for ER and 18 for PR, with the majority occurring in the low positive score range, highlighting the need for further refinement in this category. Conversely, the AI algorithm identified two true-positive ER cases that were missed by all the pathologists. This capability highlights the potential of the algorithm to significantly influence patient treatment decisions by detecting subtle positive findings that could otherwise be overlooked. This ability of the algorithm to detect critical diagnostic errors demonstrates its potential for improving diagnostic accuracy. Studies have shown improved intra- and interobserver agreement by providing pathologists with computer-aided IHC measurements during the visual scoring process. It is possible that the pathologist missed some positive cells because of the sheer size of the images. ^21^ Since consensus scoring by experts is impractical in routine practice, automated IHC measurements may provide a means to improve scores. Shafi et al. demonstrated increased efficiency in the ER assessment of breast cancer by integrating DIA in the workflow of pathologists. ^26^ AI analyzer could be used as an aid to pathologists as a ‘second reader’ in harmonizing judgments that may diverge due to over- or underestimations. ^26^ Jung et al. suggested utilizing the AI analyzer as a tool for second opinions, where pathologists can maintain their original workflow, requiring reinterpretation in only a selected subset of cases (approximately 10% for ER and PR). ^25^

Our algorithm is designed to operate on digitized whole-slide images generated through standard digital pathology workflows. Following slide scanning, the AI automatically analyzes the tumour region and generates ER and PR scores, which can then be reviewed and verified by the pathologist. The optimized two-stage architecture reduces computational time, making the workflow feasible for laboratories equipped with digital pathology infrastructure.

Limitations: One of the drawbacks of the study was the scope of validation of the AI algorithm, which was primarily confined to enhancing the concordance of pathologist interpretations. Clinical validation, such as its impact on patient survival outcomes, was not performed. Multicentre studies were included in the next version involving diverse patient populations, scanners, and staining protocols to establish the generalizability and robustness of the algorithm, however was a limitation in this study as external data sets were not used.

The version of the algorithm presented in the paper did not incorporate a tissue-type classification module to distinguish invasive carcinoma from DCIS or normal breast acini. Based on the observed misclassifications, the model is being further trained using extensive annotations to recognize and exclude ER/PR-positive normal breast acini and DCIS from analysis. Additionally, the next version will allow users to manually deselect these regions before re-running the algorithm to obtain ER and PR scores for invasive tumor alone.

### Conclusion

The algorithm showed good agreement in scoring ER and PR expressions with the ground truth, making it a reliable tool for aiding diagnostic decision-making. It can serve as a valuable support tool for pathologists and provide a second opinion, particularly in low-positive cases where human error might occur. Notably, the algorithm identified two cases in which patients could benefit from anti-estrogen treatment, highlighting its potential clinical impact.

### Ethical considerations

The study has been approved by the Kasturba Medical College and Kasturba Hospital Institutional Ethics Committee (IEC) (Ref: IEC No 05/2020 dated 18 ^th^ June 2020 and extension (Amendment) IEC No 186/2021, dated 10 ^th^ Feb 2021).

## Consent

As the study is retrospective does not involve any intervention of subjects and uses lab based coded data collection; Consent waived by the ethics committee.

## Data Availability

The dataset supporting the findings of this study, including ER and PR scores generated by both algorithmic assessment and pathologists, is publicly available in the Fig share repository at https://doi.org/10.6084/m9.figshare.31313629. ^29^ The dataset is licensed under a Creative Commons Attribution 4.0 International License (CC BY 4.0).
