# Supplementary material for: Leveraging Artificial Intelligence (AI) Based Algorithm for Accurate Estrogen Receptor (ER) and Progesterone Receptor (PR) Analysis in Breast Cancer Diagnostics: Potential to be a Crucial Aid in Routine Workflow
Source: F1000Res. 2026 Aug 3;15:702. Originally published 2026 May 11. [Version 2] doi: 10.12688/f1000research.175934.2 (PMC13560799; doi:10.12688/f1000research.175934.2)
Supplement: Peer review history

# Reviewer report: Reviewer response for version 2

**DOI:** 10.5256/f1000research.206638.r508239

**Published:** 2026-08-12

**Authors:** Yudi Kurniawan Budi Susilo

**Affiliations:** 1 University of Cyberjaya, Cyberjaya, Malaysia

This study provides a solid, practical contribution to digital pathology by addressing one of the most persistent challenges in breast cancer diagnostics: reducing inter-observer variability and standardizing immunohistochemistry scoring. 

 By evaluating the in-house "Mimansa" algorithm against an expert pathologist consensus across nearly 300 whole-slide images, the authors offer a clear and objective look at where multi-class tissue segmentation excels and where it still requires refinement. 

 The multi-stage pipeline—combining U-Net for region-of-interest segmentation with Mask R-CNN for nuclear detection—is technically sound, and testing the model on an independent cohort ensures the results are robust rather than overfitted.

 The paper's greatest strength lies in its transparency regarding performance across different expression levels. 

 While the algorithm performs exceptionally well in clear-cut negative and strongly positive cases, its lower concordance in low-positive samples mirrors the exact grey areas where human pathologists also struggle. 

 The authors do a commendable job detailing specific failure modes, such as false positives caused by normal acini, ductal carcinoma in situ, or cytoplasmic staining artifacts. 

 Addressing these limitations by incorporating an automated tissue-type classification module to filter out benign structures will be a logical and impactful next step for future iterations.

 Ultimately, the manuscript makes a compelling case for integrating AI as a supportive "second reader" in routine clinical workflows rather than a standalone diagnostic tool. 

 Highlighting how the tool successfully flagged true-positive cases initially missed by human readers underscores its immediate practical value in catching subtle malignancies. 

 To build on these promising findings, future research should focus on validating the model across multi-center datasets with diverse slide scanners and staining protocols to ensure broad generalizability. 

 Overall, the work is methodologically rigorous, well-structured, and suitable for indexing with only minor technical refinements.

Is the work clearly and accurately presented and does it cite the current literature?

Yes

If applicable, is the statistical analysis and its interpretation appropriate?

Yes

Are all the source data underlying the results available to ensure full reproducibility?

Yes

Is the study design appropriate and is the work technically sound?

Yes

Are the conclusions drawn adequately supported by the results?

Yes

Are sufficient details of methods and analysis provided to allow replication by others?

Partly

Reviewer Expertise:

AI, machine learning and deep learning.

I confirm that I have read this submission and believe that I have an appropriate level of expertise to confirm that it is of an acceptable scientific standard.

# Reviewer report: Reviewer response for version 2

**DOI:** 10.5256/f1000research.206638.r507383

**Published:** 2026-08-06

**Authors:** Chiman Haydar Salh

**Affiliations:** 1 Erbil Polytechnic University, Erbil, Iraq

Thank you for sending the revised manuscript titled '[Leveraging Artificial Intelligence (AI) Based Algorithm for Accurate Estrogen Receptor (ER) and Progesterone Receptor (PR) Analysis in Breast Cancer Diagnostics: Potential to be a Crucial Aid in Routine Workflow]'. I appreciate the authors' efforts addressing the points raised in my initial review".

 Summary of the Changes

 I summarize the key changes the authors have made, especially the most significant ones.

 "In reviewing the revised manuscript, I note that the authors have made several important changes, including [Abstract, Introduction, Related Work, Methodology, and Results and Discussion]. These revisions improve the clarity and quality of the manuscript."

Is the work clearly and accurately presented and does it cite the current literature?

Partly

If applicable, is the statistical analysis and its interpretation appropriate?

Yes

Are all the source data underlying the results available to ensure full reproducibility?

Partly

Is the study design appropriate and is the work technically sound?

Yes

Are the conclusions drawn adequately supported by the results?

Yes

Are sufficient details of methods and analysis provided to allow replication by others?

Yes

Reviewer Expertise:

NA

I confirm that I have read this submission and believe that I have an appropriate level of expertise to confirm that it is of an acceptable scientific standard.

# Reviewer report: Reviewer response for version 2

**DOI:** 10.5256/f1000research.206638.r507384

**Published:** 2026-08-05

**Authors:** Amjad Rehman

**Affiliations:** 1 Prince Sultan University, Riyadh, Saudi Arabia

Approved for indexing.

Is the work clearly and accurately presented and does it cite the current literature?

Yes

If applicable, is the statistical analysis and its interpretation appropriate?

Partly

Are all the source data underlying the results available to ensure full reproducibility?

Yes

Is the study design appropriate and is the work technically sound?

Partly

Are the conclusions drawn adequately supported by the results?

Partly

Are sufficient details of methods and analysis provided to allow replication by others?

Partly

Reviewer Expertise:

rMedical image analysis and cancer diagnosis

I confirm that I have read this submission and believe that I have an appropriate level of expertise to confirm that it is of an acceptable scientific standard.

# Reviewer report: Reviewer response for version 1

**DOI:** 10.5256/f1000research.193957.r498018

**Published:** 2026-07-14

**Authors:** Amjad Rehman

**Affiliations:** 1 Prince Sultan University, Riyadh, Saudi Arabia

This manuscript presents a well-designed study evaluating an AI-based algorithm (Mimansa) for ER and PR scoring in breast cancer IHC, demonstrating high concordance with expert pathologists and outperforming junior pathologists, with the notable advantage of identifying two true-positive ER cases missed by all pathologists.

 Several minor technical aspects warrant attention:

 (1) the model development section mentions training on "10 million patches extracted from 513 WSI from multiple data sources," but does not specify the training-validation-test split used for algorithm development or whether the 297 ER and 293 PR cases used for evaluation were part of the training data—this is critical for assessing the risk of overfitting and should be clarified;

 (2) the ground truth (final consensus score) was derived from pathologist review of discordant cases, but the manuscript does not specify the number of cases reviewed or the criteria for resolving disagreements beyond "any discrepancy was reviewed to obtain a consensus score" describing whether a third senior pathologist was involved or how tie-breaks were handled would improve transparency;

 (3) the algorithm showed discordance in 16 low-score ER cases (4 false negatives, 10 false positives, 2 true positives missed by all pathologists), but the manuscript does not report whether these 16 cases were re-reviewed by all four pathologists to confirm the algorithm's correctness—this validation step would strengthen the claim that the algorithm identified two cases missed by all pathologists;

 (4) the reason for misclassification table (Table 3) lists "Positive staining of normal breast acini" and "DCIS positive while invasive tumour negative" as causes of false positives, but the manuscript does not describe whether the algorithm currently incorporates a tissue-type classification module to distinguish invasive tumor from DCIS or normal acini.

 Lastly, comparisons, analysis, literature review with existing methods are limited and do not adequately represent recent state-of-the-art approaches. I suggest these references to be consider in the paper please

 [Ref 1]

 2. Kurdi, S. Z. (2026). Machine Learning–Based Classification Framework for Human Health Care Monitoring. International Journal of Theoretical & Applied Computational Intelligence, 2026, Vol. 2026, 1–15. https://doi.org/10.65278/IJTACI.2026.1

 3. Mughal, B., Muhammad, N., Sharif, M., Saba, T., & Rehman, A. (2017). Extraction of breast border and removal of pectoral muscle in wavelet domain. Biomedical Research, 28(11), 5041-5043.

 4. Abdzaid, A. Y., Khadum, D. N., Al-Barrak, S. S., & Saad, A. (2026). White Blood Cells Classification Based on a New Strategy to Cluster Channels RGB of Blood Smear Image Using Resnet-18 Model. International Journal of Theoretical & Applied Computational Intelligence, 2026, 120-131.

 [Ref 5]

Is the work clearly and accurately presented and does it cite the current literature?

Yes

If applicable, is the statistical analysis and its interpretation appropriate?

Partly

Are all the source data underlying the results available to ensure full reproducibility?

Yes

Is the study design appropriate and is the work technically sound?

Partly

Are the conclusions drawn adequately supported by the results?

Partly

Are sufficient details of methods and analysis provided to allow replication by others?

Partly

Reviewer Expertise:

rMedical image analysis and cancer diagnosis

I confirm that I have read this submission and believe that I have an appropriate level of expertise to confirm that it is of an acceptable scientific standard, however I have significant reservations, as outlined above.

## References

[1] : Feature Selection Methods in Big Medical Databases:A Comprehensive Survey. *International Journal of Theoritical & Applied Computational Intelligence* .2025; 10.65278/IJTACI.2025.21181-209 10.65278/IJTACI.2025.21

[2] : Cloud‐based decision support system for the detection and classification of malignant cells in breast cancer using breast cytology images. *Microscopy Research and Technique* .2019;82(6) : 10.1002/jemt.23222775-785 10.1002/jemt.23222 30697861

# Response

**Published:** 2026-07-16

**Authors:** Kanthilatha Pai

**Affiliations:** Pathology, Kasturba Medical College Manipal, Manipal, Karnataka, India

Thank you for sparing your valuable time to review our article. We appreciate time and effort in providing feedback on our manuscript and are grateful for the insightful comments.everal minor technical aspects warrant attention:

 Reviewer (1) the model development section mentions training on "10 million patches extracted from 513 WSI from multiple data sources," but does not specify the training-validation-test split used for algorithm development or whether the 297 ER and 293 PR cases used for evaluation were part of the training data—this is critical for assessing the risk of overfitting and should be clarified;

 Response: The 513 WSI were used for model development (training, validation and testing). The 297 ER and 293 PR cases were separate data set that were used to test the developed algorithm

 Reviewer(2) the ground truth (final consensus score) was derived from pathologist review of discordant cases, but the manuscript does not specify the number of cases reviewed or the criteria for resolving disagreements beyond "any discrepancy was reviewed to obtain a consensus score" describing whether a third senior pathologist was involved or how tie-breaks were handled would improve transparency;

 Response: The consensus score was obtained by joint review of all discordant cases by the four pathologists using a penta-head microscope. Discrepancies were resolved through discussion to obtain a final consensus score (ground truth). No third independent pathologist was involved, as all disagreements were resolved through this consensus review.

 Reviewer (3) the algorithm showed discordance in 16 low-score ER cases (4 false negatives, 10 false positives, 2 true positives missed by all pathologists), but the manuscript does not report whether these 16 cases were re-reviewed by all four pathologists to confirm the algorithm's correctness—this validation step would strengthen the claim that the algorithm identified two cases missed by all pathologists;

 Response:The algorithm score was compared with the initial (pathologist) consensus score, and any discordance was reviewed through joint discussion by the four pathologists to obtain a final consensus score (ground truth) 

 Reviewer(4) the reason for misclassification table (Table 3) lists "Positive staining of normal breast acini" and "DCIS positive while invasive tumour negative" as causes of false positives, but the manuscript does not describe whether the algorithm currently incorporates a tissue-type classification module to distinguish invasive tumor from DCIS or normal acini.

 Response: The version of the algorithm presented in the paper does not incorporate a tissue-type classification module to distinguish invasive carcinoma from DCIS or normal breast acini. Based on the observed misclassifications, the model is being further trained using extensive annotations to recognize and exclude ER/PR-positive normal breast acini and DCIS from analysis. Additionally, the next version will allow users to manually deselect these regions before re-running the algorithm to obtain ER and PR scores for invasive tumor alone.

 Reviewer : Lastly, comparisons, analysis, literature review with existing methods are limited and do not adequately represent recent state-of-the-art approaches. I suggest these references to be consider in the paper please

 Response: The mentioned references will be included in the revised version in the discussion

 Artificial intelligence (AI) has increasingly been applied across various domains of medical imaging, including image reconstruction, processing, analysis, and predictive modelling.

 Reference: Kurdi, S. Z. (2026). Machine Learning–Based Classification Framework for Human Health Care Monitoring. International Journal of Theoretical & Applied Computational Intelligence, 2026, Vol. 2026, 1–15. https://doi.org/10.65278/IJTACI.2026.1

 In breast cancer, AI technologies have been widely adopted for early detection through AI-assisted radiology and for diagnostic assessment and biomarker evaluation in digital pathology.

 Reference:  Mughal, B., Muhammad, N., Sharif, M., Saba, T., & Rehman, A. (2017). Extraction of breast border and removal of pectoral muscle in wavelet domain. Biomedical Research, 28(11), 5041-5043.

 Appropriate image preprocessing and augmentation strategies have been shown to improve feature extraction and enhance the performance of deep learning models in medical image analysis.

 Reference : Abdzaid, A. Y., Khadum, D. N., Al-Barrak, S. S., & Saad, A. (2026). White Blood Cells Classification Based on a New Strategy to Cluster Channels RGB of Blood Smear Image Using Resnet-18 Model. International Journal of Theoretical & Applied Computational Intelligence, 2026, 120-131.

# Reviewer report: Reviewer response for version 1

**DOI:** 10.5256/f1000research.193957.r486492

**Published:** 2026-06-16

**Authors:** Chiman Haydar Salh

**Affiliations:** 1 Erbil Polytechnic University, Erbil, Iraq

The manuscript is well written, addresses an important clinical application of AI in digital pathology, and presents results that support the conclusions. The study demonstrates high concordance between the AI algorithm and expert pathologist assessment, indicating that the algorithm may serve as a valuable second-reader tool. The manuscript is suitable for indexing after minor revisions aimed at improving methodological transparency and reproducibility.

 The manuscript clearly demonstrates that the algorithm performs well in strongly positive and negative cases. The discussion appropriately acknowledges the reduced performance in low-positive cases and identifies potential causes of false-positive and false-negative classifications.

 While the overall methodology is described, the manuscript would benefit from additional technical details regarding the AI model architecture, training strategy, validation procedures, and performance optimization. Providing more information would improve reproducibility and allow independent evaluation of the algorithm.

 Consider including additional information regarding external validation on datasets from other institutions to assess generalizability.

 The discussion could further elaborate on how the identified sources of error (normal acini, DCIS, tissue folds, staining artefacts) may be addressed in future versions of the algorithm.

 A brief discussion of computational requirements and workflow integration in routine pathology laboratories would enhance the practical relevance of the study.

Is the work clearly and accurately presented and does it cite the current literature?

Partly

If applicable, is the statistical analysis and its interpretation appropriate?

Yes

Are all the source data underlying the results available to ensure full reproducibility?

Partly

Is the study design appropriate and is the work technically sound?

Yes

Are the conclusions drawn adequately supported by the results?

Yes

Are sufficient details of methods and analysis provided to allow replication by others?

Yes

Reviewer Expertise:

NA

I confirm that I have read this submission and believe that I have an appropriate level of expertise to confirm that it is of an acceptable scientific standard, however I have significant reservations, as outlined above.

# Response

**Published:** 2026-07-16

**Authors:** Kanthilatha Pai

**Affiliations:** Pathology, Kasturba Medical College Manipal, Manipal, Karnataka, India

Thank you for sparing your valuable time to review our article. We appreciate time and effort in providing feedback on our manuscript and are grateful for the insightful comments.

 Reviewer : While the overall methodology is described, the manuscript would benefit from additional technical details regarding the AI model architecture, training strategy, validation procedures, and performance optimization. Providing more information would improve reproducibility and allow independent evaluation of the algorithm.

 Response :The AI pipeline employed a two-stage architecture. A U-Net-based segmentation model was used initially to identify tumour regions within whole-slide images, restricting subsequent analysis to regions of interest. Nuclei detection and classification within these tumour regions were then performed using a customized Mask R-CNN model. The algorithm was trained on manually annotated image patches generated by expert pathologists, with extensive data augmentation (rotation, translation, shearing, cropping, and colour perturbation) were used to improve robustness. Performance was evaluated at both the cell level against expert annotations and the slide level using independent datasets. The two-stage approach reduced the average processing time from approximately 30 to 12 minutes per slide while maintaining high reproducibility, with approximately 99% agreement across repeated analyses.

 Reviewer: Consider including additional information regarding external validation on datasets from other institutions to assess generalizability.

 Response: Multicentre studies were included in the next version involving diverse patient populations, scanners, and staining protocols to establish the generalizability and robustness of the algorithm, however was a limitation in this study as external data sets were not used.

 Reviewer: The discussion could further elaborate on how the identified sources of error (normal acini, DCIS, tissue folds, staining artefacts) may be addressed in future versions of the algorithm.

 Response: The version of the algorithm presented in the paper does not incorporate a tissue-type classification module to distinguish invasive carcinoma from DCIS or normal breast acini. Based on the observed misclassifications, the model is being further trained using extensive annotations to recognize and exclude ER/PR-positive normal breast acini and DCIS from analysis. Additionally, the next version will allow users to manually deselect these regions before re-running the algorithm to obtain ER and PR scores for invasive tumor alone.

 Reviewer: A brief discussion of computational requirements and workflow integration in routine pathology laboratories would enhance the practical relevance of the study.

 Response: The algorithm is designed to operate on digitized whole-slide images generated through standard digital pathology workflows. Following slide scanning, the AI automatically analyzes the tumour region and generates ER and PR scores, which can then be reviewed and verified by the pathologist. The optimized two-stage architecture reduces computational time, making the workflow feasible for laboratories equipped with digital pathology infrastructure. As emphasized in the paper, the AI is intended as a decision-support tool to improve efficiency and consistency while maintaining pathologist oversight
